# SMARCA4-deficient NSCLC treated with first-line tislelizumab and fruquintinib achieved remarkable tumor regression: case report and literature review

**DOI:** 10.3389/fimmu.2025.1521828

**Published:** 2025-04-17

**Authors:** Jie Dong, Jinli Zhang, Hongming Pan, Chongwei Wang, Jin Sheng

**Affiliations:** ^1^ Department of Medical Oncology, Sir Run Run Shaw Hospital, School of Medicine, Zhejiang University, Zhejiang, China; ^2^ Department of Pathology, Sir Run Run Shaw Hospital, School of Medicine, Zhejiang University, Hangzhou, China

**Keywords:** SMARCA4-deficient NSCLC, targeted therapy, immunotherapy, case report, precision oncology

## Abstract

SMARCA4-deficient non-small cell lung cancer (SMARCA4-dNSCLC) typically lacks target-driven gene alterations and are primarily resistant to cytotoxic drugs. There is currently no standard treatment, especially for those who are unwilling or unable to receive chemotherapy. This case reported that chemotherapy-free strategy with tislelizumab and fruquintinib was utilized as a first-line treatment for a patient with SMARCA4-deficient NSCLC, and the patient achieved remarkable partial remission and lasted more than two years of disease control without severe adverse events.

## Introduction

SMARCA4-dNSCLC usually lacks targetable sensitive gene mutations and are primarily resistant to multiple cytotoxic drugs (1). These patients have relatively poor prognosis and lack standard treatment (2). Most patients will receive chemotherapy or immune checkpoint inhibitors combined with chemotherapy (3). For patients who are unwilling or unfit for chemotherapy, there is no standard treatment plan. Currently, studies have explored the first-line treatment of NSCLC patients with immune checkpoint inhibitors (ICIs) combined with anti-angiogenesis agents. Preliminary findings suggest that this combination therapy exhibits promising efficacy and tolerability (4). This case reported that tislelizumab combined with fruquintinib utilized as the first-line treatment for patients with SMARCA4-dNSCLC. In the initial efficacy assessment, the patient experienced partial remission of the lesion. Unfortunately, cervical lymph node progression occurred. The diagnosis confirmed the same pathological type via a subsequent biopsy. The patient was subsequently treated with a combination of local radiotherapy and previous systemic therapy. Despite this, the patient achieved more than two years of disease control without grade ≥3 adverse events.

## Case description

**Figure 1:**
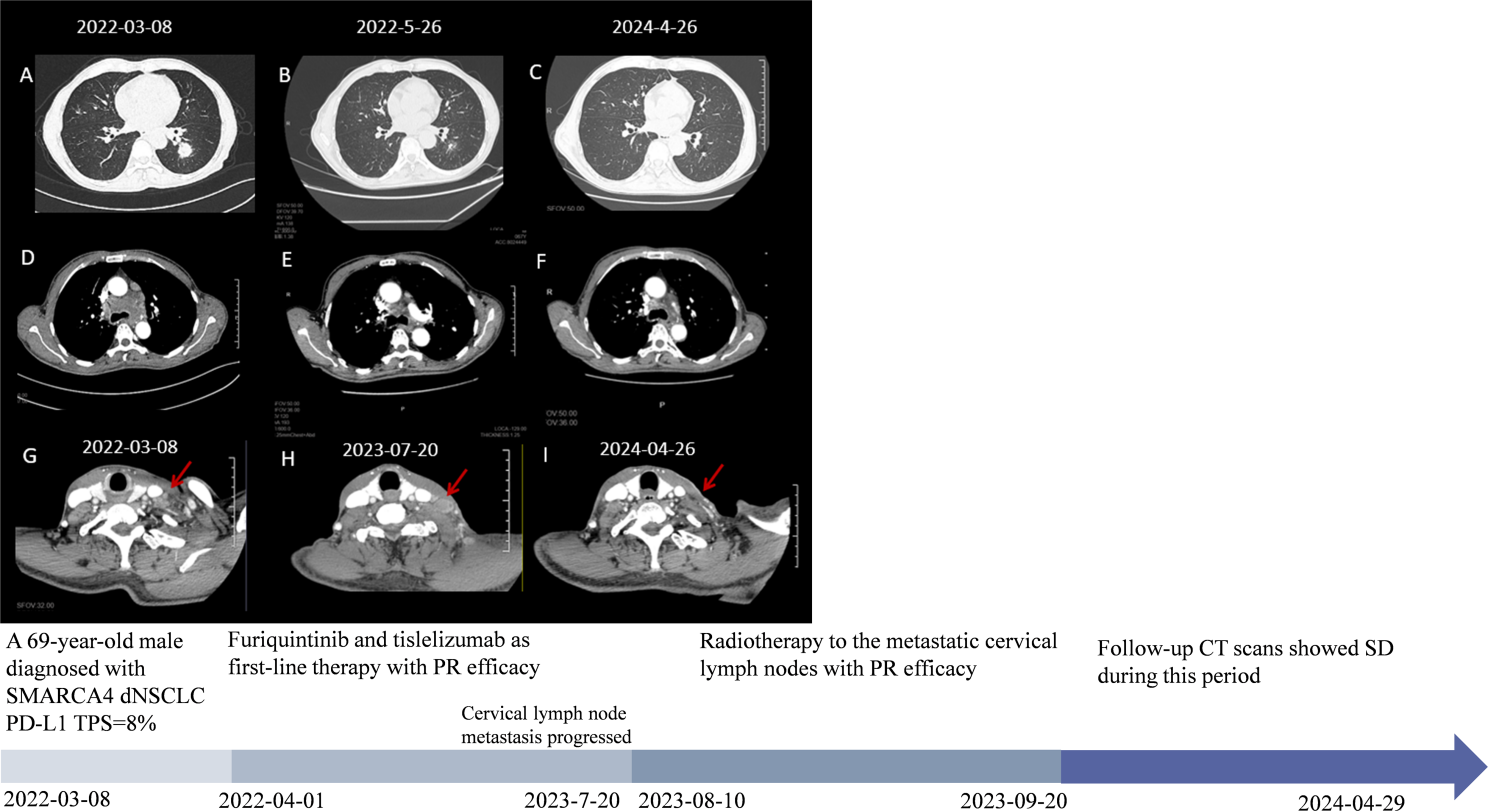
The patient’s clinical course schedule. **(A, D, G)** Computed tomography scans of lesions at baseline before tislelizumab and fruquintinib treatment; **(B, E)** CT represents scans for partial response (PR) efficacy after two cycles of tislelizumab and fruquintinib; **(C, F)** Latest chest CT scans and **(G, H, I)** disease changes of cervical lymph node metastasis during treatment. A detailed timeline encapsulates the key milestones, data points, and relevant clinical context from the patient’s episode of care.

**Figure 2:**
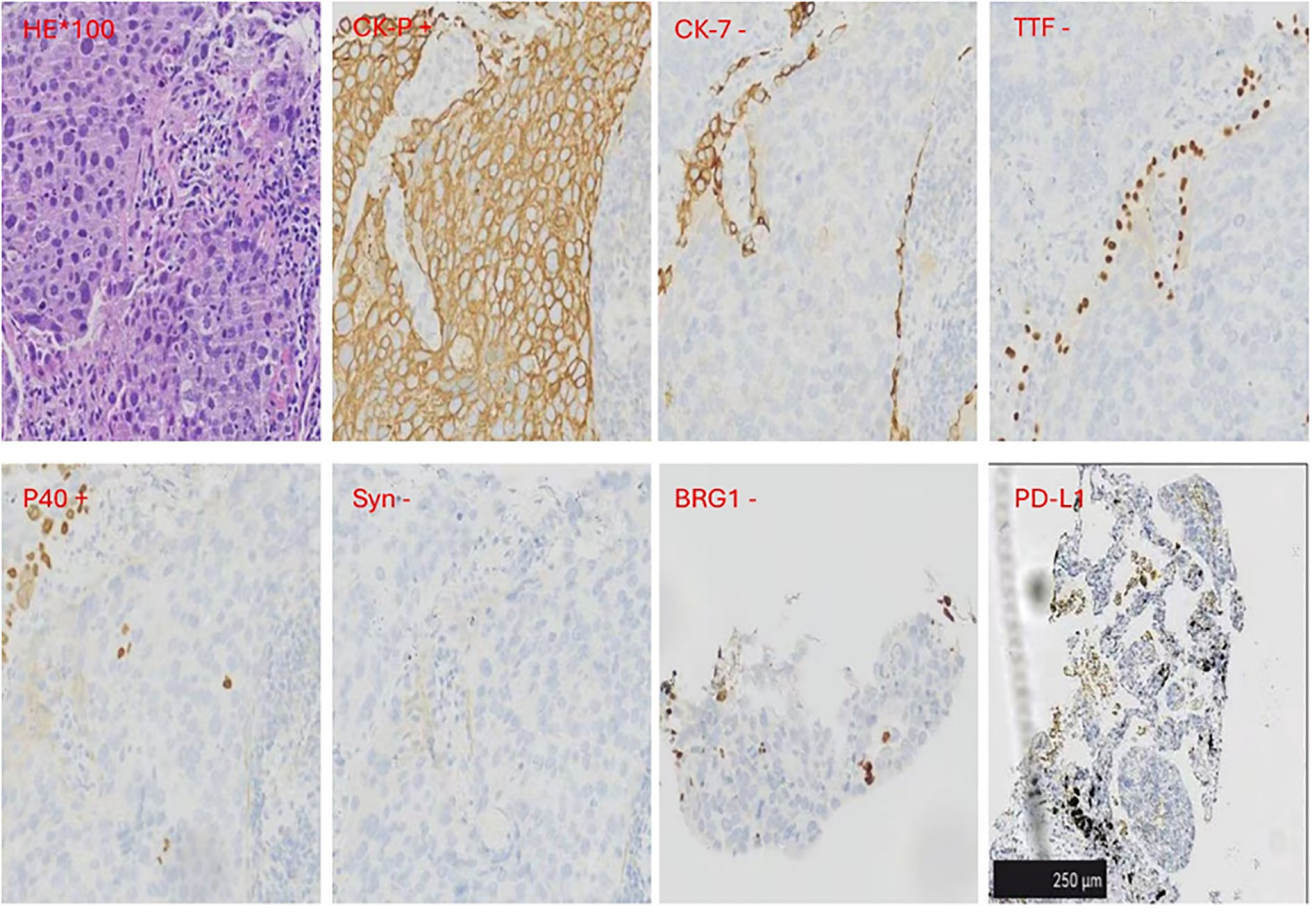
Immunohistochemical (IHC) results. IHC markers were CK-pan and P40 positive.CK7, TTF-1, BRG-1and Syn were negative. The Dako PD-L1 22C3 assay immunohistochemistry assay for PD-L1 expression revealed that the tumor proportion score (TPS) was 8%. Scale bar=250μm.

In February 2022, a 67-year-old man with ECOG PS 1 who had smoked 1 pack per day for 40 years presented to our hospital for chest tightness. The patients reported no significant past medical history, family history, or psychosocial history. Physical examination showed left supraclavicular lymphadenopathy, about 1 cm in size. Chest CT examination revealed a mass of about 2 cm in size in the posterior basal segment of the left lower lobe, with multiple burrs on the edge and enhancement. Multiple lymph nodes in the mediastinum were enlarged and fused, surrounding the left main bronchus (**Figures 1A, D, G**). To further clarify the diagnosis, a CT-guided lung puncture biopsy was performed on March 10, 2022. Preliminary pathology suggested poorly differentiated carcinoma. As shown in **Figure 2**, immunohistochemical (IHC) markers were CK-pan (+), CK7 (–), TTF-1 (–), P40 (+), BRG-1 (–) and Syn (–). Combined with IHC results, it was considered to be SMARCA4-dNSCLC, and PD-L1 staining revealed a tumor proportion score (TPS) of 8% (**Figure 2**). Second-generation gene detection of tumor tissue did not reveal any target-sensitive mutations. The patient’s abdominal enhanced CT showed enlargement of the left adrenal gland, which was considered to be a metastatic lesion. Emission computed tomograph (ECT) whole-body bone imaging and brain enhanced MRI showed no obvious abnormalities.

The patient preferred to join a chemotherapy-free clinical trial. Therefore, he was screened and enrolled in an “Efficacy and Safety of Tislelizumab in Combination with Fruquintinib in Participants with Selected Solid Tumors (ClinicalTrials.gov ID, NCT04716634)” at Sir Run Run Shaw Hospital (SRRSH), School of Medicine, Zhejiang University.

The patient was then treated with furiquintinib (5 mg for three weeks and one week off) combined with tislelizumab (300 mg q4w) since April 1, 2022. The tumor was partially relieved at the first follow-up examination and remained stable during subsequent treatment (**Figures 1B, C, E, F**). On May 12, 2022, due to pain in both upper limbs, which affected daily life, he stopped taking furiquintinib on his own. The pain improved after stopping the medication. On May 27, 2022, he received a reduced dose of furiquintinib (4 mg orally once daily for 21 consecutive days, followed by a 7-day rest period to complete each 28-day cycle) combined with tislelizumab (300mg q4w). On June 2023, the patient developed pain in the cervical lymph node area and the cervical lymph nodes were enlarged compared to the previous examination. To rule out pseudoprogression during immunotherapy, the patient was re-examined on July 2023, and the cervical lymph nodes are still enlarged (**Figure 1H**). Considering the progression of metastasis of cervical lymph nodes, lymph node re-biopsies were performed and results still showed SMARCA4-dNSCLC (**Supplementary Figure 1**). From August 10, 2023 to September 20, 2023, along with the previous treatment, he received radiotherapy to the metastatic cervical lymph nodes: GTV (left cervical enlarged lymph nodes) 6, 10mv-X SAD 100DT 6600cGy/30F/42d, CTV (left cervical high-risk area) 6, 10mv-X SAD 100DT 6000cGy/30F/42d. After radiotherapy, the patient’s neck metastasis was significantly reduced and pain improved (**Figure 1I**). On 26 April 2024, the last imaging review was conducted, which showed stable disease (**Figures 1C, F, I**). The last clinical drug was on April 29, 2024, after which he took the initiative to apply for termination of treatment for being in the clinical trial project for two years and the patient was subsequently followed up regularly. Throughout the treatment process, the main adverse reaction observed were grade 2 oral mucositis and grade 2 secondary hypothyroidism, which were given symptomatic treatment.

## Discussion

The *SMARCA4* has been identified as a tumor suppressor gene, located on chromosome 19p13, encoding the BRG1 protein, which is an important component of the SWI/SNF chromatin remodeling complex, which has transcriptional regulation and DNA damage repair functions. Loss of BRG1 protein can lead to dysfunction of the above complex, resulting in the occurrence and development of various cancers (5–7). Continuous loss of BRG1 protein expression in tumor cells can lead topathological diagnosis. SMARCA4-deficient thoracic tumors are currently divided into two main categories: SMARCA4-dNSCLC and SMARCA4-deficient undifferentiated carcinoma (8). Recent studies have shown that NSCLC patients with *SMARCA4* mutations (especially homozygous deletions and truncated mutations, which result in loss of BRG protein) are more likely to develop drug resistance, early tumor relapse, and poor prognosis than *SMARCA4* wild-type patients (9, 10). Mutation analysis of the *SMARCA4* gene revealed a unique pattern of mutations that were mutually exclusive with the most common target oncogene mutations in non-small cell lung cancer (NSCLC), such as *EGFR*, *ALK*, *MET*, *ROS1*, and *RET*. This finding was consistent with the genetic testing results of this patient. However, *SMARCA4* mutations may also be associated with smoking-related mutations such as *KRAS*, *STK11*, and *KEAP1* (2, 11).

**Figure 3:**
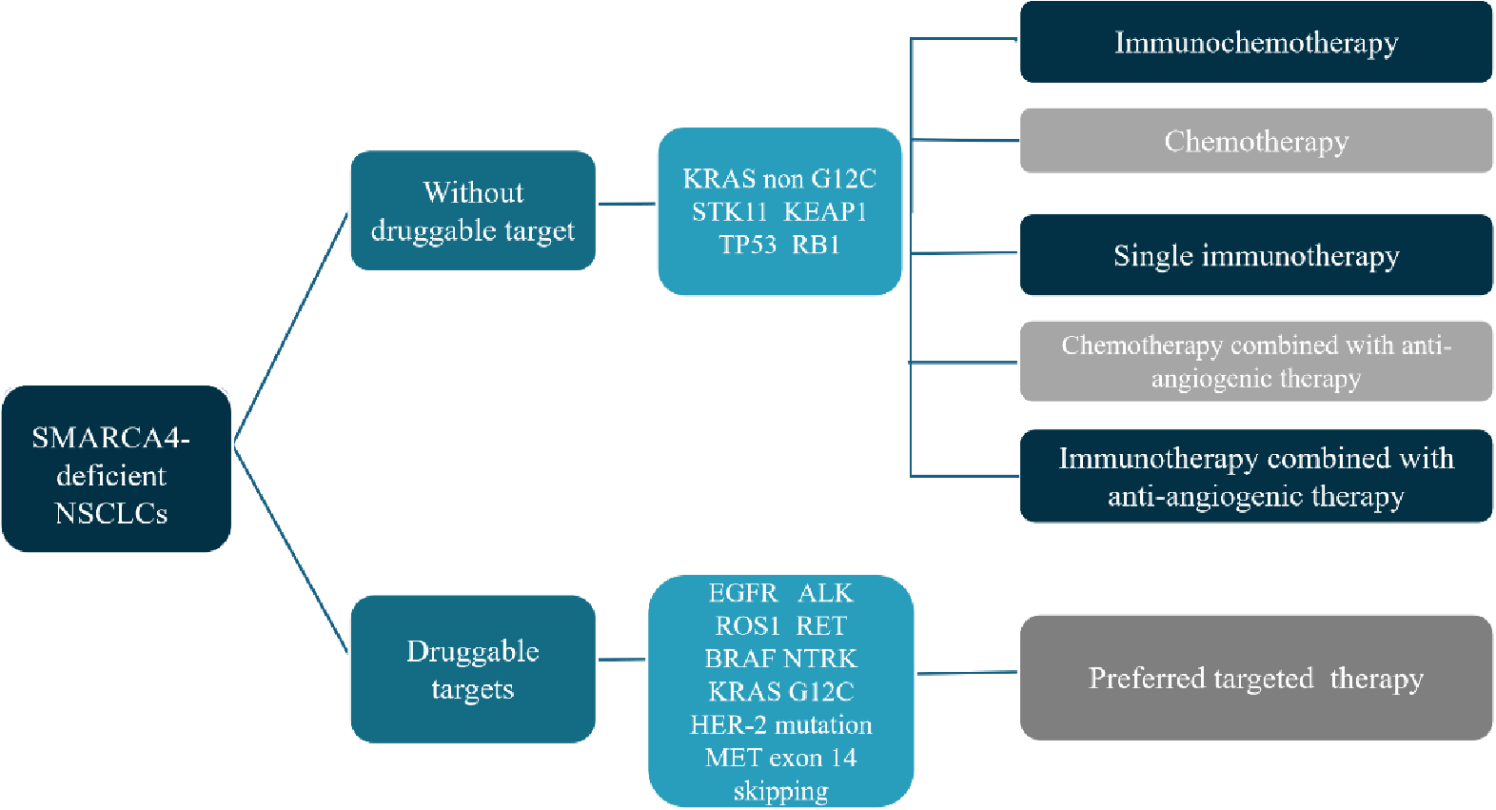
Treatment strategies proposed for SMARCA4-dNSCLC.

The mutation rate of *SMARCA4* in NSCLC is about 10% and can be divided into two categories: Category 1, including truncating mutations, fusions and homozygous deletions; Category 2, including missense mutations. Loss of SMARCA4 expression was significantly associated with category 1 variants (12). The prognosis of this group of people is relatively poor, and there is currently no standard treatment recommendation. Here we briefly discuss the treatment of SMARCA4-dNSCLC (**Figure 3**).

For this group of people, the current mainstream first-line treatment options are mainly immunotherapy combined with platinum-based chemotherapy. Compared with patients who receive chemotherapy alone, patients who receive immunotherapy combined with chemotherapy may have improved OS benefits (3, 13). Single-agent immunotherapy may also benefit some patients. For example, Tomoyuki Naito et al. reported a SMARCA4-dNSCLC with negative PD-L1 expression and a relatively high TMB of 396 mutations. The fourth-line treatment used single-agent nivolumab, and disease control has been maintained for more than 14 months (14). In addition, there was also a report of an advanced SMARCA4-dNSCLC with high PD-L1 expression of 50%-60%, who received pembrolizumab for second line treatment, and the best efficacy reached complete response (15). Joao V. Alessi et al. retrospectively analyzed the survival of 532 patients of SMARCA4-dNSCLC who received immunotherapy for advanced disease. Unfortunately, there was no statistical difference in ORR and median PFS and OS between SWI/SNF wild-type and mutant NSCLC in their study. This indicated that the efficacy of immunotherapy for SWI/SNF mutant NSCLCs were no less than that of wild-type patients (11). However, there was also study inconsistent with this result (16). In addition, a large retrospective study cohort studies had shown that SMARCA4-deficient patients may have a higher response rate to immune checkpoint inhibitors; however, no survival benefit was observed (12). Hence, single-agent immunotherapy may be a treatment option for SMARCA4-dNSCLC and may not be dependent on PD-L1 expression. The TMB value of SMARCA4-dNSCLC is higher than that of wild-type patients, but it may not be related to the efficacy of immunotherapy (17). Therefore, we still need large-scale clinical studies to further explore the exact efficacy of single-agent immunotherapy in SMARCA4-dNSCLC and screen appropriate efficacy predictors. In addition to immunotherapy alone, combined therapy with ICI, chemotherapy and anti-angiogenic drugs may also have a certain therapeutic effect in SMARCA4-dNSCLC (18).

SMARCA4-dNSCLCs are often accompanied by gene mutations such as *KRAS*, *STK11, TP53, KEAP1*, and *LRP1B*, and rarely by gene mutations such as *EGFR, ALK*, and *ROS1 (* 9). Hence, few reports are on gene-targeted therapy, and the effect is generally poor. For example, the case reported by Xiyue Liang et al. showed that NSCLC patients with *SMARCA4* mutations combined with *EGFR* exon21 L858R missense mutations only achieved four months of disease control with first-line osimertinib. Another NSCLC patient was SMARCA4 deletion combined with *ROS1* fusion mutation, and received first-line treatment with pemetrexed combined with carboplatin and ensartinib, with an overall survival of only 3.7 months (19). In patients with *SMARCA4* mutations, *KRAS, STK11, or KEAP1* mutations are common, but these mutations may be associated with resistance to immunotherapy (11, 20, 21).

For patients who are unwilling to receive chemotherapy or cannot tolerate chemotherapy, ICIs combined with anti-vascular targeted drugs may also be a beneficial option for patients. In this case, tislelizumab combined with fruquintinib was used as the first-line treatment, which provided the patient with disease control for more than two years. During this case study, our focus was primarily on SMARCA4 IHC status, system treatment implementation, clinical response, and safety management. Of course, this case failed to explore the genetic status of *SMARCA4* in terms of genetics, which is also a shortcoming of this case.

## Conclusion

This case reports the combination of tislelizumab and fruquintinib as first-line treatment for a patient with *SMARCA4*-deficient NSCLC. After cervical lymph node progression, local radiotherapy was combined, which provided the patient with more than two years of disease control and provided a potential first-line treatment option for patients who are unwilling to receive or intolerant of chemotherapy.

## Data Availability

The original contributions presented in the study are included in the article/**Supplementary Material**. Further inquiries can be directed to the corresponding author.
