# Supplementary material for: SMARCA4-deficient NSCLC treated with first-line tislelizumab and fruquintinib achieved remarkable tumor regression: case report and literature review
Source: Front Immunol. 2025 Apr 17;16:1521828. doi: 10.3389/fimmu.2025.1521828 (PMC12043563; doi:10.3389/fimmu.2025.1521828)
Supplement: Supplementary file 1 [file Data Sheet 1.PDF › Peer Review Only/Data Sheet 1.PDF]

SIR RUN RUN SHAW HOSPITAL, MEDICAL SCHOOL, ZHEJIANG UNIVERSITY
浙江大学医学院附属邵逸夫医院
MOLECULAR PATHOLOGY DIAGNOSTIC CENTER
分子病理诊断中心
GENE MUTATION DETECTION REPORT
基因突变检测报告单

Molecular Accession No.（分子病理编号）：**ARMS-LMG-2022-00086**

Name（姓名）：[redacted] **Sex**（性别）：男 **Age**（年龄）：**67**岁 **Med.Rec.No.**（病历号）：[redacted]

Pathological No.（病理号）：**xsB2022-06374B** **Content of Tumor Cells%**（肿瘤细胞比例%）：**70%**

Sample Type（标本类型）：活检标本石蜡切片 **Sampling Date**(采样日期)：**2022-03-10**

Date Requested（申请日期）：**2022-03-11** **Date Received**（接收日期）：**2022-03-11**

Requested by（科室/医生/电话）：[redacted]

Clinical Diagnosis（临床诊断）：肺占位性病变

Pathological Diagnosis（病理诊断）：详见病理报告单

Result（结果）： 未发现该试剂盒检测范围内的突变。

检测项目	外显子/密码子	突变类型	检测结果
EGFR基因	Exon-19	19-del	野生型
	Exon-21	L858R	野生型
	Exon-20	T790M	野生型
	Exon-20	20-ins	野生型
	Exon-20	S768I	野生型
	Exon-18	G719X	野生型
	Exon-21	L861Q	野生型
KRAS基因	Exon-2	G12D/S	野生型
		G12A/V/R/C、G13C	野生型
BRAF基因	Exon-15	V600E1/E2/K/R/D1/D2	野生型
NRAS基因*	Exon-3	Q61R/K/L/H	野生型
HER2基因*	Exon-20	A775_G776insYVMA/M774_A775insAYVM	野生型
		/G776>VC/ P780_Y781insGSP/ G776>LC	野生型
MET 基因*	Exon-13<br>Exon-15	Exon-14<br>跳跃突变	野生型
PIK3CA基因*	Exon-20	H1047R	野生型
	Exon-9	E545K	

检测项目	外显子/密码子	突变类型	检测结果
ALK融合基因	ALK-Exon-20	E13;A20,E6ins33;A20，E20;A20，E18;A20，E2;A20，<br>E17;ins68A20,E2;ins117A20、<br>E13;ins69A20、E6;A20、E6;A19、<br>E6;ins18A20、E20;ins18A20、<br>E17del58;ins39A20、E17ins65;A20、<br>E17;ins30A20、E17ins61;ins34A20、<br>E3;ins53A20、KI24;A20、KI17;A20、KL9;A20、<br>T4;A20	野生型
ROS1融合基因	ROS1-Exon-32/34	SLC34A2 Exon-4/14、<br>CD74 Exon-6、SDC4 Exon-2/4<br>SLC34A2 Exon-4/14、EZR Exon-10、<br>CD74 Exon-6、SDC4 Exon-4	野生型
	ROS1-Exon-35	TPM3 Exon-8、<br>LRIG3 Exon-16、GOPC Exon-8	野生型
RET融合基因*	RET-Exon-12	CCDC6 exon 1;RET exon 12<br>NCOA4 exon 6;RET exon 12<br>KIF5B exon 15;RET exon 12<br>KIF5B exon 16;RET exon 12<br>KIF5B exon 23;RET exon 12<br>KIF5B exon 22;RET exon 12	野生型

备注：*表示此本试剂盒中的该突变位未达到临床试剂盒注册要求，结果仅供参考。

Operator（操作人）：[redacted]

Reporter（报告人）：

Verifier（审核人）：

Date/ Time（报告日期）： 2022-03-14

Instructions（说明）：

1，Methods（检测方法）：本试剂盒采用荧光定量PCR方法（ADX-ARMS法），可分别检测患者组织DNA/RNA样本中人类EGFR、ALK、ROS1、KRAS、BRAF基因突变，此检测范围外的突变无法检测或未达到临床试剂盒注册要求，结果仅供参考。如果检测样本的肿瘤细胞含量低于5%，可能会影响检测结果的准确性。
2，Reagents（试剂）：5种突变基因检测试剂盒（厦门艾德生物医药科技股份有限公司）。
3，Analysis Instrument（分析仪器）：SLAN-96S（宏石，中国）。

Annotation（附注）：Molecular Pathological Diagnosis must combine the clinical and surgical pathological diagnosis. This report is valid only for the checked tissue. Any questions, please contact us!
（分子病理诊断必须结合临床及常规病理所见而给予解释，此报告仅对送检测组织有效。如有疑问请及时与我们联系）

Address（地址）：3 East Qingchun Road，Hangzhou，Zhejiang，310016（浙江省杭州市 庆春东路3号310016）
Tel （电话）：0571-86002270

MOLECULAR PATHOLOGY REPORT

Patient Name: [redacted]

Med.Rec.No.：
