# Supplementary material for: SMARCA4-deficient NSCLC treated with first-line tislelizumab and fruquintinib achieved remarkable tumor regression: case report and literature review
Source: Front Immunol. 2025 Apr 17;16:1521828. doi: 10.3389/fimmu.2025.1521828 (PMC12043563; doi:10.3389/fimmu.2025.1521828)
Supplement: Supplementary file 1 [file Data Sheet 2.PDF › Peer Review Only/Data Sheet 2.PDF]

打印时间:2024/10/29 10:01:07

浙江大学医学院附属邵逸夫医院

病理检查报告单(二次报告)

病理序号： B2023-45038

姓　名：	[redacted]	性别：	男	年龄：	68岁	取材日期：	2023-07-21
病历号：		床号：	012061			送检科室：	肿瘤内科

检查项目： 常规组织病理学检查（活检标本）

病理所见：

免疫组化 B2023-45038：
A片：CK-pan(AE1/AE3)（+）、CK7（部分+）、TTF-1（-）、NapsinA（-）、CK5/6（-）、P63（-）、P40（-）、BRG1（-）、NUT（-）、Ki-67（80%+）。

病理诊断：

（左侧锁骨上淋巴结穿刺）低分化癌，结合免疫组化检测结果，倾向SMARC4缺失性癌

备 注：

2023-07-27补充免疫组化结果，完成二次报告

书写医生：[redacted]　　审核医生：[redacted]　　报告日期：2023-07-27

病理检查报告单

PATHOLOGY REPORT

姓　名：[redacted]

病历号：

打印时间:2024/10/29 10:01:05

浙江大学医学院附属邵逸夫医院

病理检查报告单(二次报告)

病理序号： **xsB2022-06374**

姓　名：	[redacted]	性别：	男	年龄：	67岁	取材日期：	2022-03-10
病历号：	[redacted]	床号：	612E29			送检科室：	微创介入XS

检查项目： xs常规组织病理学检查（活检标本）

病理所见：

免疫组化 xsB2022-06374：
B片：CK-pan(AE1/AE3)（+）、CK5/6（+）、P40（部分+）、CK7（-）、TTF-1（-）、NapsinA（-）、Syn（-）、CgA（-）、Ki-67（40%+）
A片：PD-L1(22C3)（阳性，TPS=8%）（判读标准参考非小细胞肺癌TPS标准，结果仅供临床参考）；
免疫组化 xsB2022-06374：
2023-08-14 补充 A片+B片：BRG1（部分-）、NUT（-）

病理诊断：

（左肺穿刺）非小细胞癌，结合免疫组化，考虑低分化鳞状细胞癌

备 注：

书写医生：[redacted]　　审核医生：[redacted]　　报告日期：2022-03-18

病理检查报告单

PATHOLOGY REPORT

姓　名：[redacted]

病历号：[redacted]
