# Supplementary material for: SMARCA4-deficient NSCLC treated with first-line tislelizumab and fruquintinib achieved remarkable tumor regression: case report and literature review
Source: Front Immunol. 2025 Apr 17;16:1521828. doi: 10.3389/fimmu.2025.1521828 (PMC12043563; doi:10.3389/fimmu.2025.1521828)
Supplement: Supplementary file 1 [file DataSheet1.zip › Peer Review Only/Supplementary Figure 1.DOCX]

**Supplementary Figure 1.** Immunohistochemical staining for the patient in cervical lymph node metastasis (100×).
